# Supplementary figures and images for: RBMX2 links Mycobacterium bovis infection to epithelial–mesenchymal transition and lung cancer progression (part 1 of 2)
Source: eLife. 2025 Nov 24;14:RP107132. doi: 10.7554/eLife.107132 (PMC12643470; doi:10.7554/eLife.107132)

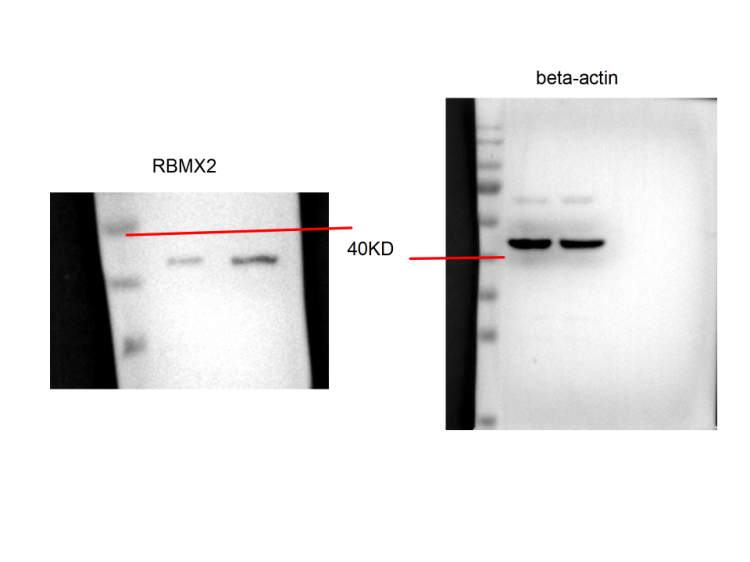

Supplement: Figure 1—source data 1. [file elife-107132-fig1-data1.zip › Figure 1 source data1-1/ALL.tif]

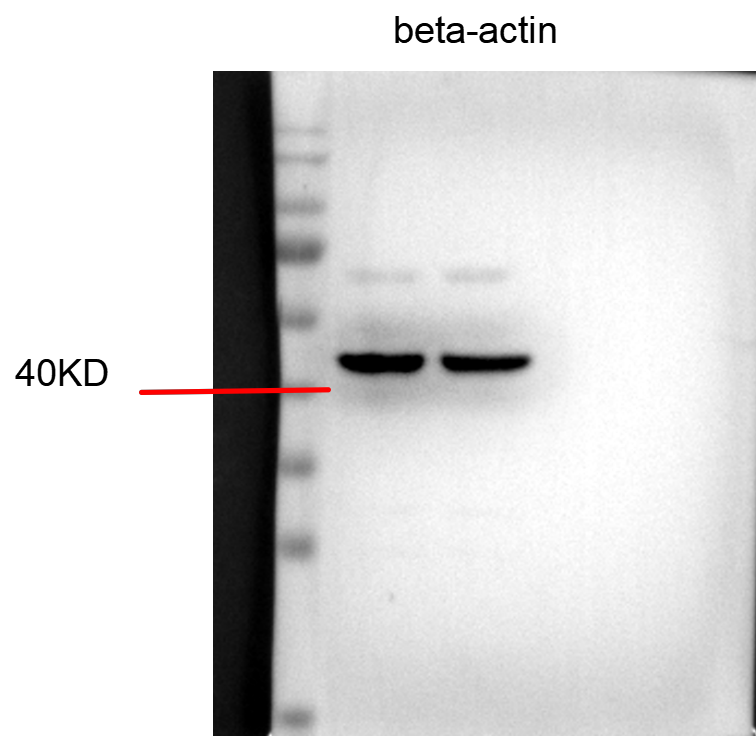

Supplement: Figure 1—source data 1. [file elife-107132-fig1-data1.zip › Figure 1 source data1-1/BA.tif]

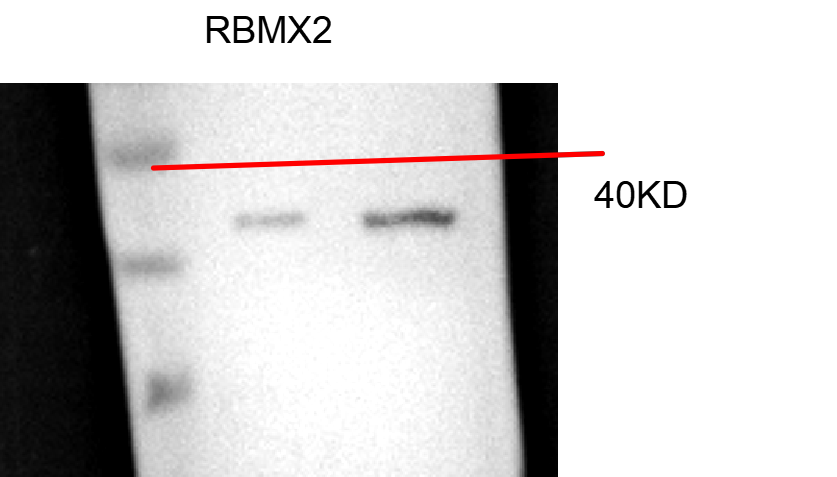

Supplement: Figure 1—source data 1. [file elife-107132-fig1-data1.zip › Figure 1 source data1-1/RB.tif]

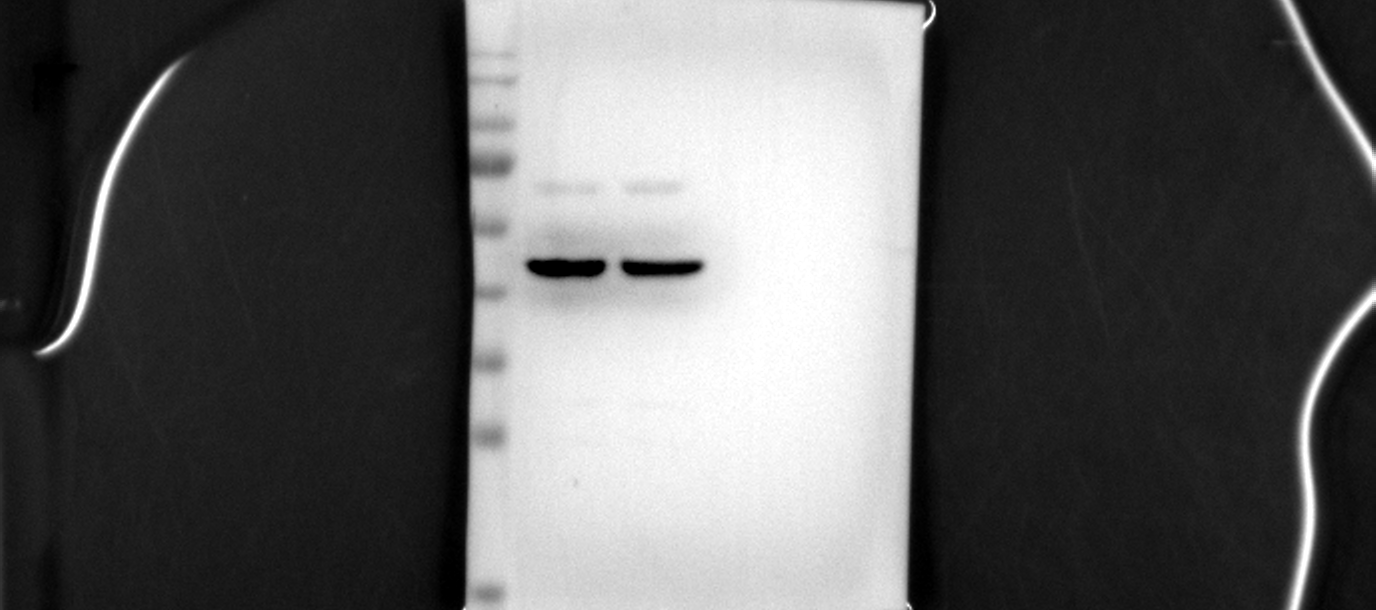

Supplement: Figure 1—source data 2. [file elife-107132-fig1-data2.zip › Figure 1 source data1/BA.png]

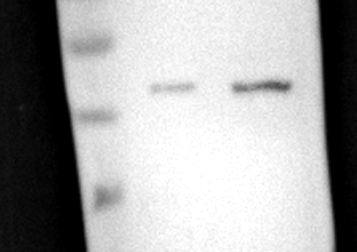

Supplement: Figure 1—source data 2. [file elife-107132-fig1-data2.zip › Figure 1 source data1/RBMX2.png]

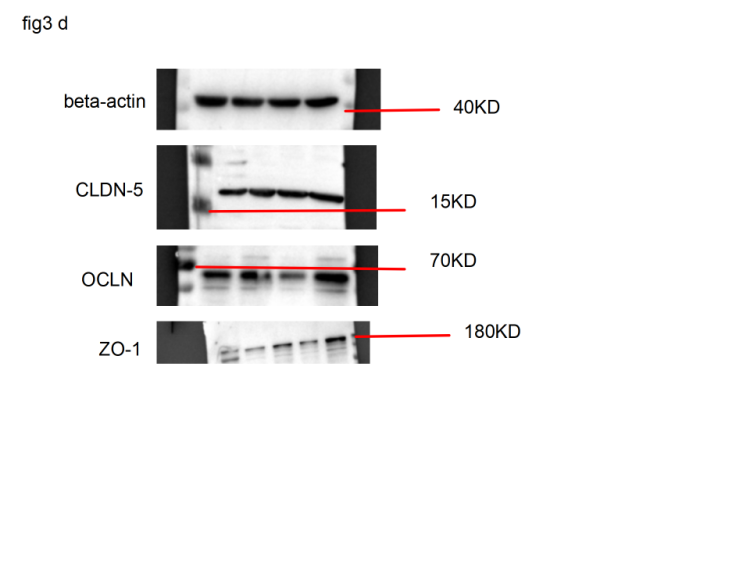

Supplement: Figure 3—source data 1. [file elife-107132-fig3-data1.zip › Figture3 source data1-1/ALL.tif]

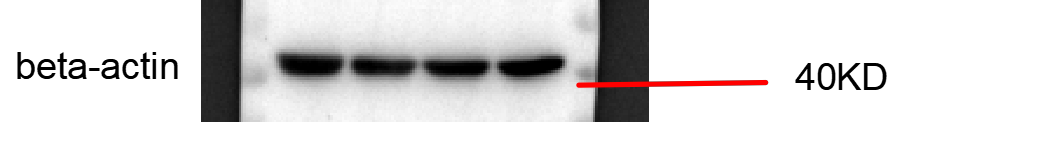

Supplement: Figure 3—source data 1. [file elife-107132-fig3-data1.zip › Figture3 source data1-1/ba.tif]

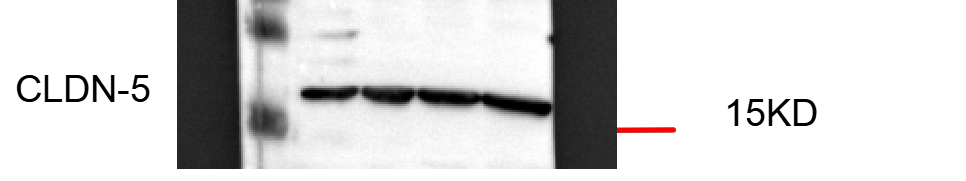

Supplement: Figure 3—source data 1. [file elife-107132-fig3-data1.zip › Figture3 source data1-1/C5.tif]

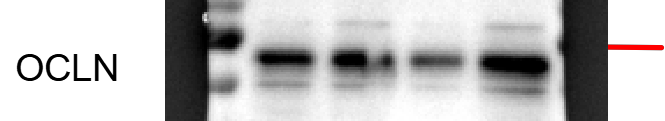

Supplement: Figure 3—source data 1. [file elife-107132-fig3-data1.zip › Figture3 source data1-1/OCLN1.tif]

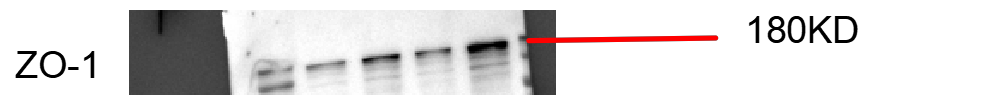

Supplement: Figure 3—source data 1. [file elife-107132-fig3-data1.zip › Figture3 source data1-1/ZO1.tif]

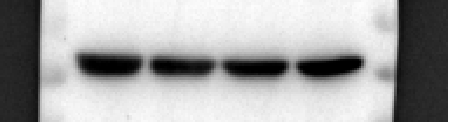

Supplement: Figure 3—source data 2. [file elife-107132-fig3-data2.zip › Figure 3 source data1/BA.tif]

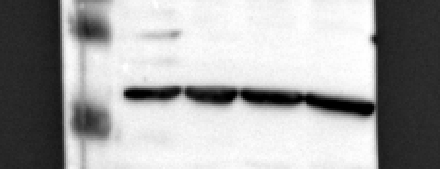

Supplement: Figure 3—source data 2. [file elife-107132-fig3-data2.zip › Figure 3 source data1/C5.tif]

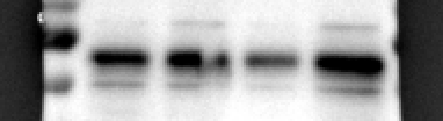

Supplement: Figure 3—source data 2. [file elife-107132-fig3-data2.zip › Figure 3 source data1/OCLN.tif]

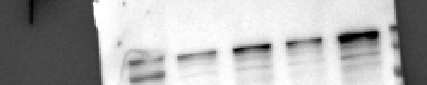

Supplement: Figure 3—source data 2. [file elife-107132-fig3-data2.zip › Figure 3 source data1/ZO1.tif]

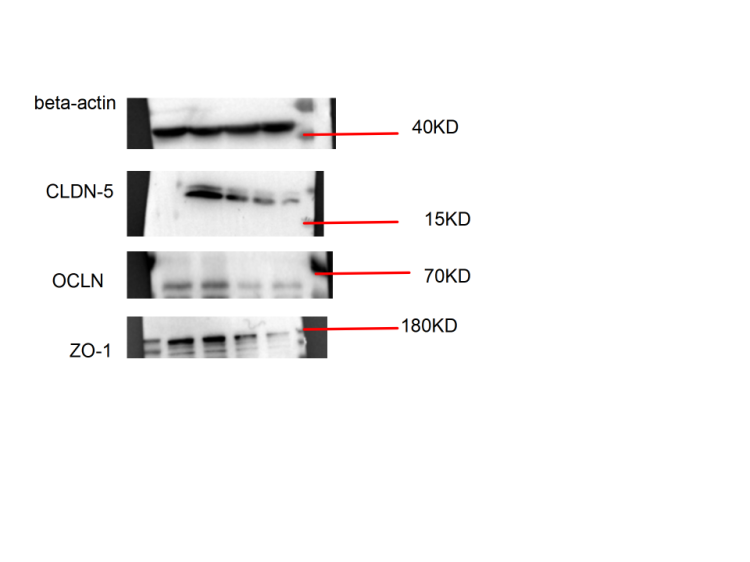

Supplement: Figure 3—source data 3. [file elife-107132-fig3-data3.zip › Figture3 source data2-1/ALL.tif]

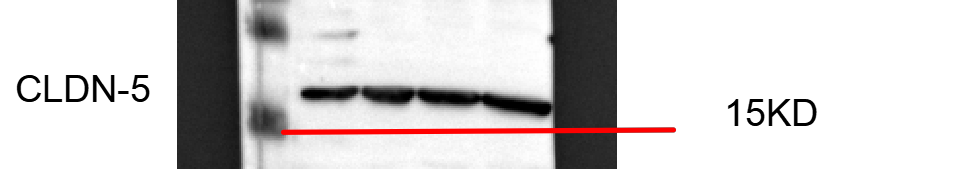

Supplement: Figure 3—source data 3. [file elife-107132-fig3-data3.zip › Figture3 source data2-1/C5.tif]

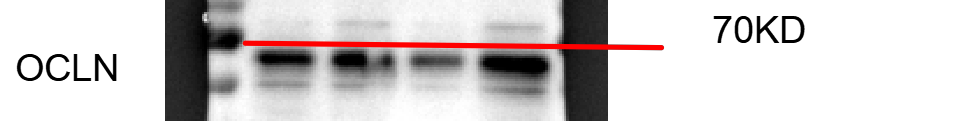

Supplement: Figure 3—source data 3. [file elife-107132-fig3-data3.zip › Figture3 source data2-1/OCLN.tif]

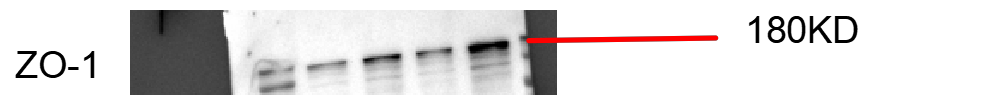

Supplement: Figure 3—source data 3. [file elife-107132-fig3-data3.zip › Figture3 source data2-1/ZO1.tif]

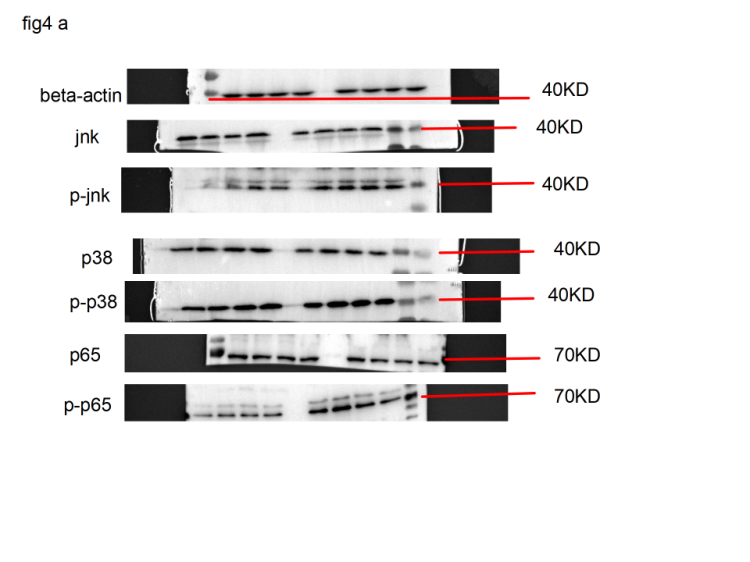

Supplement: Figure 4—source data 1. [file elife-107132-fig4-data1.zip › Figure 4 source data1-1/ALL.tif]

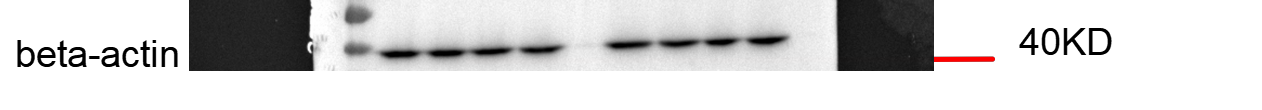

Supplement: Figure 4—source data 1. [file elife-107132-fig4-data1.zip › Figure 4 source data1-1/BA.tif]

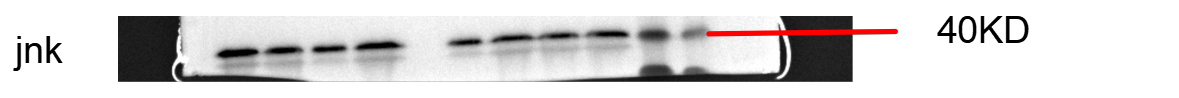

Supplement: Figure 4—source data 1. [file elife-107132-fig4-data1.zip › Figure 4 source data1-1/JNK.tif]

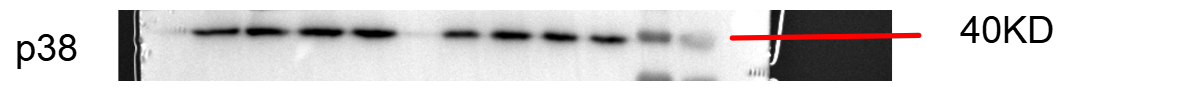

Supplement: Figure 4—source data 1. [file elife-107132-fig4-data1.zip › Figure 4 source data1-1/P38.tif]

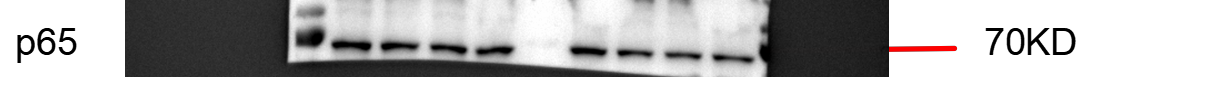

Supplement: Figure 4—source data 1. [file elife-107132-fig4-data1.zip › Figure 4 source data1-1/P65.tif]

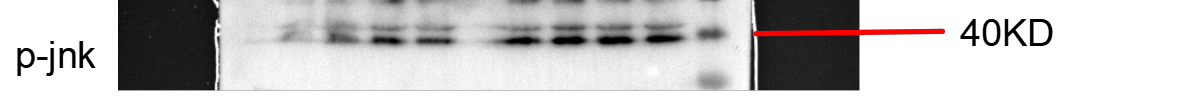

Supplement: Figure 4—source data 1. [file elife-107132-fig4-data1.zip › Figure 4 source data1-1/PJNK.tif]

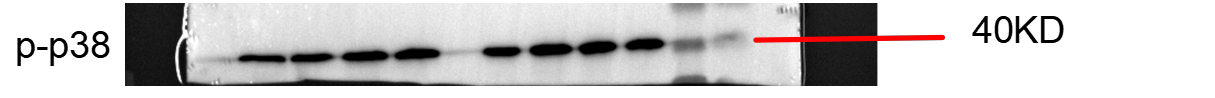

Supplement: Figure 4—source data 1. [file elife-107132-fig4-data1.zip › Figure 4 source data1-1/PP38.tif]

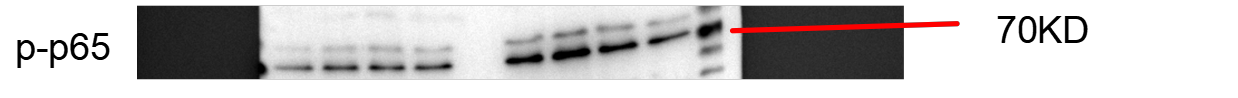

Supplement: Figure 4—source data 1. [file elife-107132-fig4-data1.zip › Figure 4 source data1-1/PP65.tif]

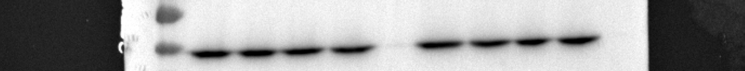

Supplement: Figure 4—source data 2. [file elife-107132-fig4-data2.zip › Figure 4 source data1/BA.tif]

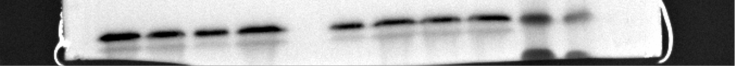

Supplement: Figure 4—source data 2. [file elife-107132-fig4-data2.zip › Figure 4 source data1/JNK.tif]

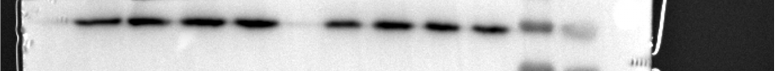

Supplement: Figure 4—source data 2. [file elife-107132-fig4-data2.zip › Figure 4 source data1/P38.tif]

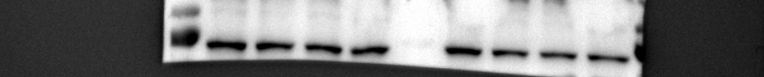

Supplement: Figure 4—source data 2. [file elife-107132-fig4-data2.zip › Figure 4 source data1/P65.tif]

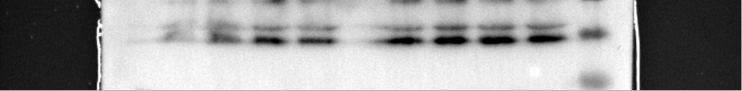

Supplement: Figure 4—source data 2. [file elife-107132-fig4-data2.zip › Figure 4 source data1/PJNK1.tif]

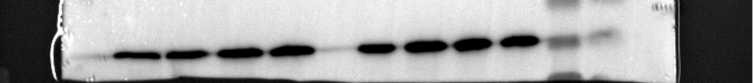

Supplement: Figure 4—source data 2. [file elife-107132-fig4-data2.zip › Figure 4 source data1/PP38.tif]

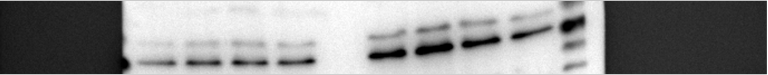

Supplement: Figure 4—source data 2. [file elife-107132-fig4-data2.zip › Figure 4 source data1/PP65.tif]

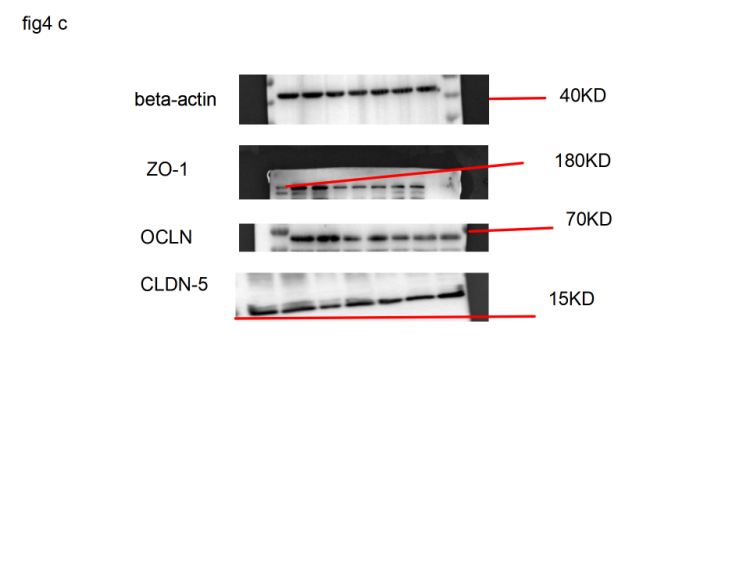

Supplement: Figure 4—source data 3. [file elife-107132-fig4-data3.zip › Figure 4 source data2-1/ALL.tif]

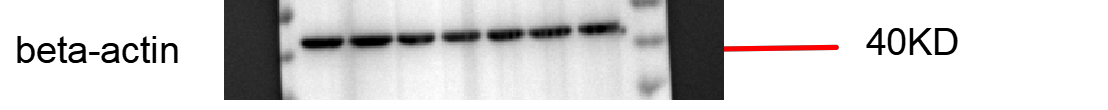

Supplement: Figure 4—source data 3. [file elife-107132-fig4-data3.zip › Figure 4 source data2-1/BA.tif]

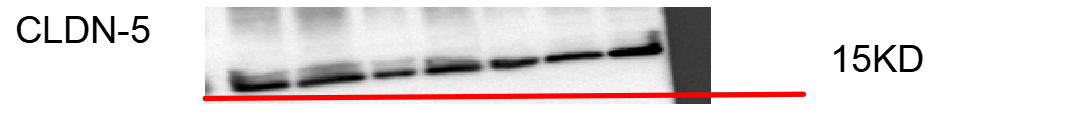

Supplement: Figure 4—source data 3. [file elife-107132-fig4-data3.zip › Figure 4 source data2-1/CLDN5.tif]

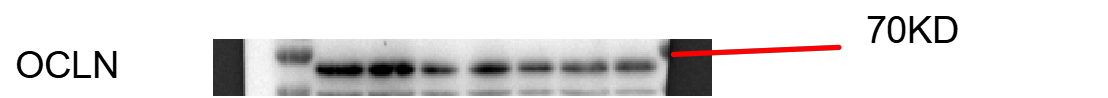

Supplement: Figure 4—source data 3. [file elife-107132-fig4-data3.zip › Figure 4 source data2-1/OCLN.tif]

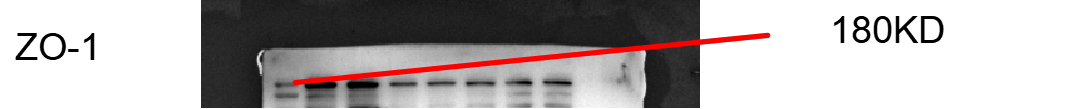

Supplement: Figure 4—source data 3. [file elife-107132-fig4-data3.zip › Figure 4 source data2-1/ZO1.tif]

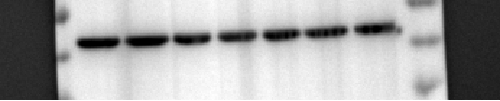

Supplement: Figure 4—source data 4. [file elife-107132-fig4-data4.zip › Figure 4 source data2/BA.tif]

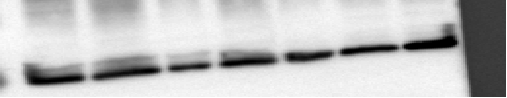

Supplement: Figure 4—source data 4. [file elife-107132-fig4-data4.zip › Figure 4 source data2/CLAN5.tif]

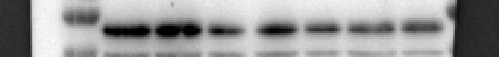

Supplement: Figure 4—source data 4. [file elife-107132-fig4-data4.zip › Figure 4 source data2/OCLN.tif]

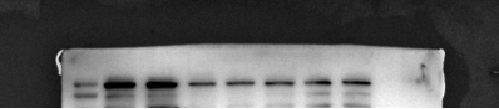

Supplement: Figure 4—source data 4. [file elife-107132-fig4-data4.zip › Figure 4 source data2/ZO1.tif]

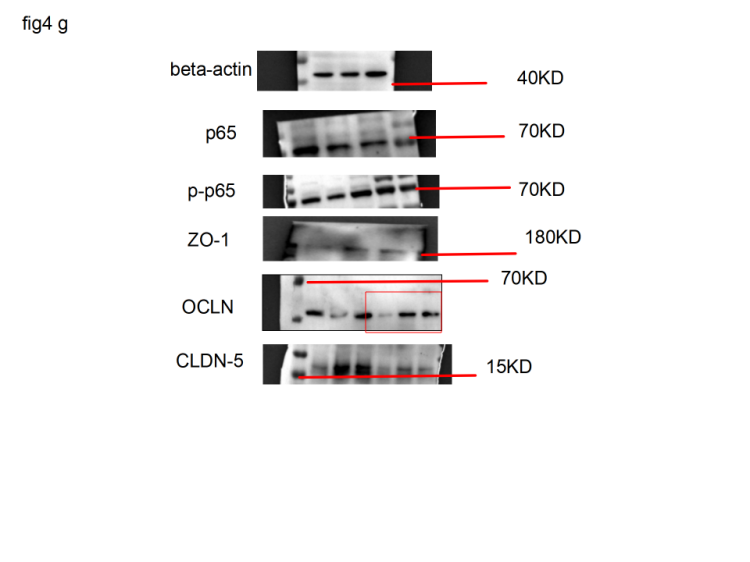

Supplement: Figure 4—source data 5. [file elife-107132-fig4-data5.zip › Figure 4 source data3-1/ALL.tif]

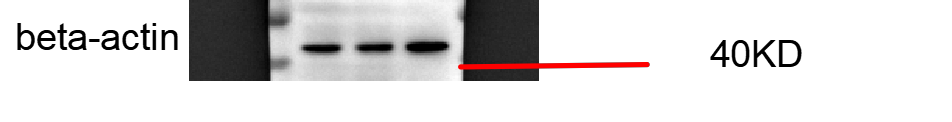

Supplement: Figure 4—source data 5. [file elife-107132-fig4-data5.zip › Figure 4 source data3-1/BA.tif]

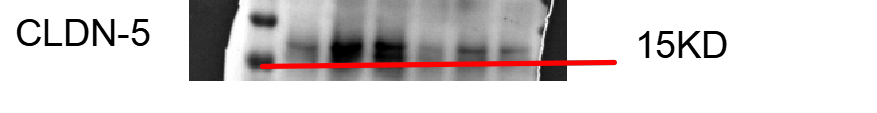

Supplement: Figure 4—source data 5. [file elife-107132-fig4-data5.zip › Figure 4 source data3-1/CLDN5.tif]

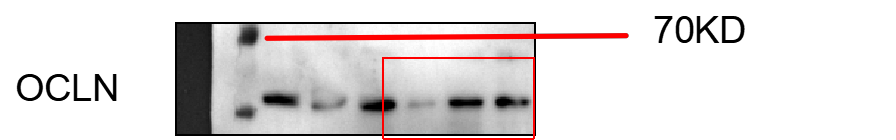

Supplement: Figure 4—source data 5. [file elife-107132-fig4-data5.zip › Figure 4 source data3-1/OCLN.tif]

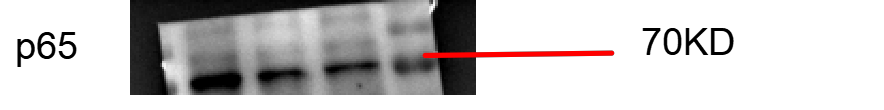

Supplement: Figure 4—source data 5. [file elife-107132-fig4-data5.zip › Figure 4 source data3-1/P65.tif]

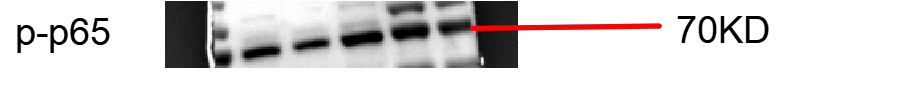

Supplement: Figure 4—source data 5. [file elife-107132-fig4-data5.zip › Figure 4 source data3-1/PP65.tif]

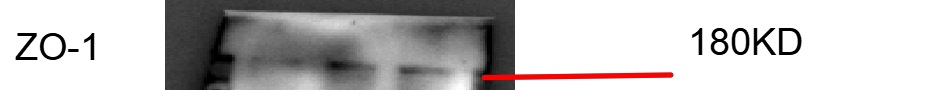

Supplement: Figure 4—source data 5. [file elife-107132-fig4-data5.zip › Figure 4 source data3-1/ZO1.tif]

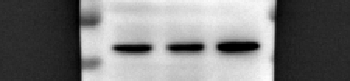

Supplement: Figure 4—source data 6. [file elife-107132-fig4-data6.zip › Figure 4 source data3/BA.tif]

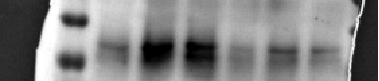

Supplement: Figure 4—source data 6. [file elife-107132-fig4-data6.zip › Figure 4 source data3/CLDN5.tif]

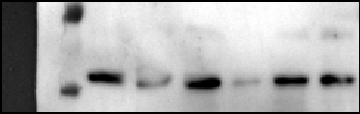

Supplement: Figure 4—source data 6. [file elife-107132-fig4-data6.zip › Figure 4 source data3/OCLN.tif]

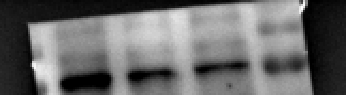

Supplement: Figure 4—source data 6. [file elife-107132-fig4-data6.zip › Figure 4 source data3/P65.tif]

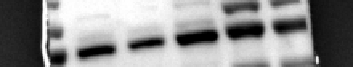

Supplement: Figure 4—source data 6. [file elife-107132-fig4-data6.zip › Figure 4 source data3/PP65.tif]

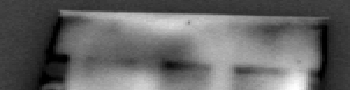

Supplement: Figure 4—source data 6. [file elife-107132-fig4-data6.zip › Figure 4 source data3/ZO1.tif]

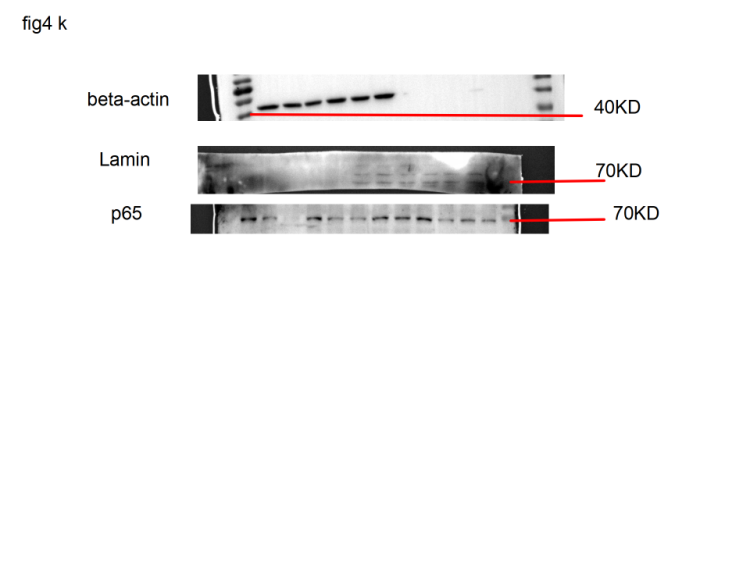

Supplement: Figure 4—source data 7. [file elife-107132-fig4-data7.zip › Figure 4 source data4-1/ALL.tif]

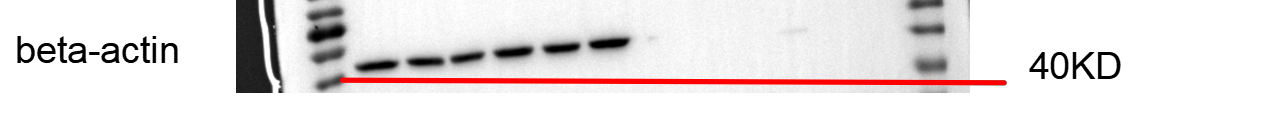

Supplement: Figure 4—source data 7. [file elife-107132-fig4-data7.zip › Figure 4 source data4-1/BA.tif]

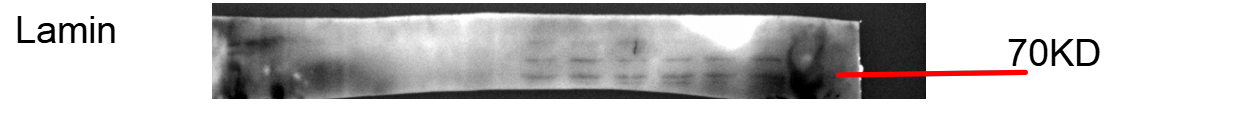

Supplement: Figure 4—source data 7. [file elife-107132-fig4-data7.zip › Figure 4 source data4-1/LAMIN.tif]

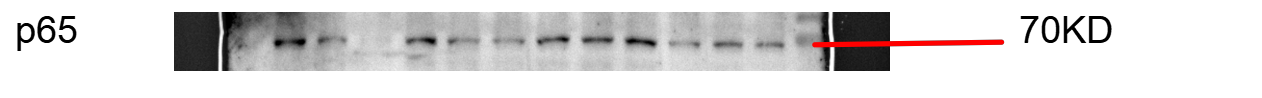

Supplement: Figure 4—source data 7. [file elife-107132-fig4-data7.zip › Figure 4 source data4-1/P65.tif]

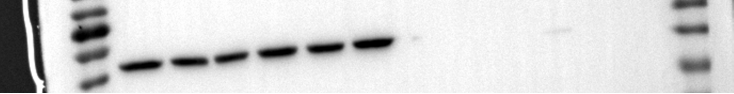

Supplement: Figure 4—source data 8. [file elife-107132-fig4-data8.zip › Figure 4 source data4/BA.tif]

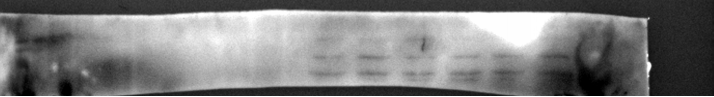

Supplement: Figure 4—source data 8. [file elife-107132-fig4-data8.zip › Figure 4 source data4/LAMIN.tif]

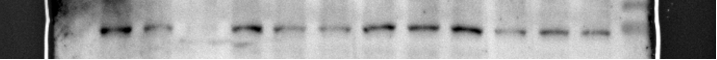

Supplement: Figure 4—source data 8. [file elife-107132-fig4-data8.zip › Figure 4 source data4/P65.tif]

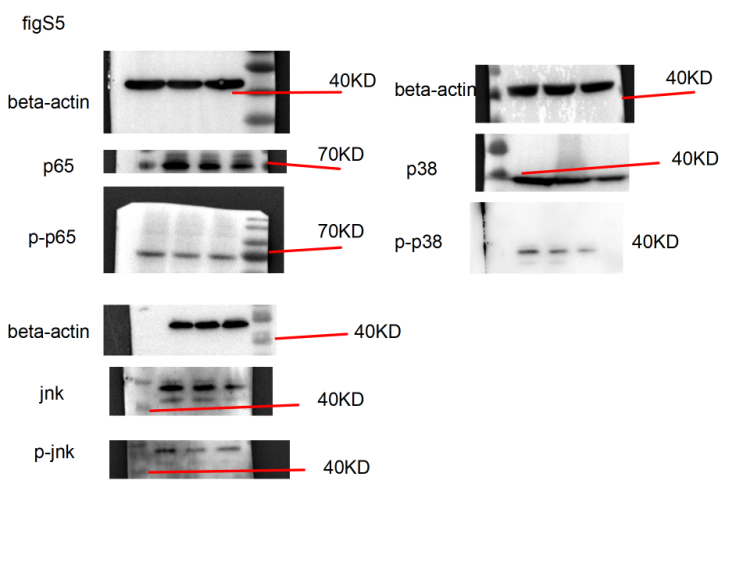

Supplement: Figure 4—figure supplement 1—source data 1. [file elife-107132-fig4-figsupp1-data1.zip › Figure 4-figure suplement 1 source data1-1/ALL.tif]

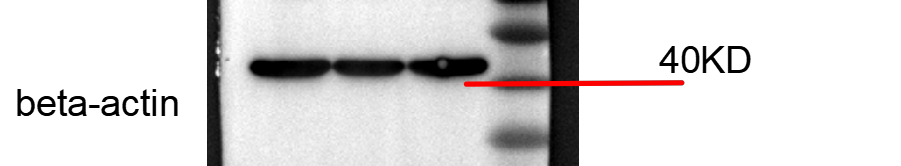

Supplement: Figure 4—figure supplement 1—source data 1. [file elife-107132-fig4-figsupp1-data1.zip › Figure 4-figure suplement 1 source data1-1/ba1.tif]

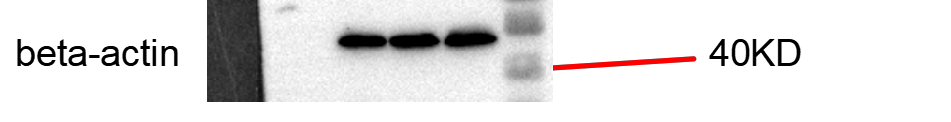

Supplement: Figure 4—figure supplement 1—source data 1. [file elife-107132-fig4-figsupp1-data1.zip › Figure 4-figure suplement 1 source data1-1/ba2.tif]

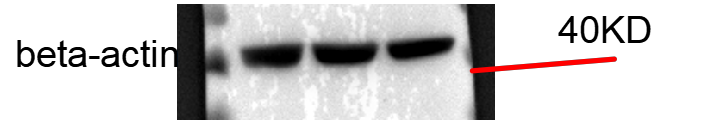

Supplement: Figure 4—figure supplement 1—source data 1. [file elife-107132-fig4-figsupp1-data1.zip › Figure 4-figure suplement 1 source data1-1/ba3.tif]

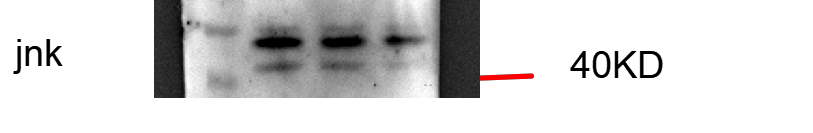

Supplement: Figure 4—figure supplement 1—source data 1. [file elife-107132-fig4-figsupp1-data1.zip › Figure 4-figure suplement 1 source data1-1/jnk.tif]

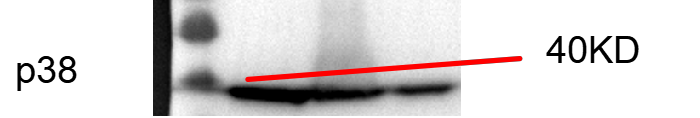

Supplement: Figure 4—figure supplement 1—source data 1. [file elife-107132-fig4-figsupp1-data1.zip › Figure 4-figure suplement 1 source data1-1/p38.tif]

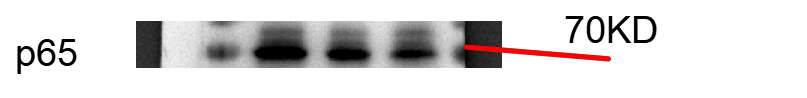

Supplement: Figure 4—figure supplement 1—source data 1. [file elife-107132-fig4-figsupp1-data1.zip › Figure 4-figure suplement 1 source data1-1/p65.tif]

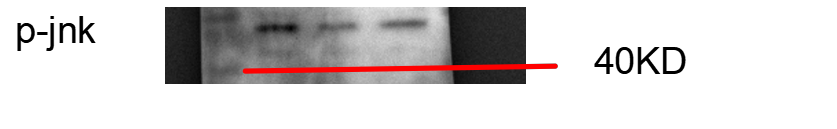

Supplement: Figure 4—figure supplement 1—source data 1. [file elife-107132-fig4-figsupp1-data1.zip › Figure 4-figure suplement 1 source data1-1/pjnk.tif]

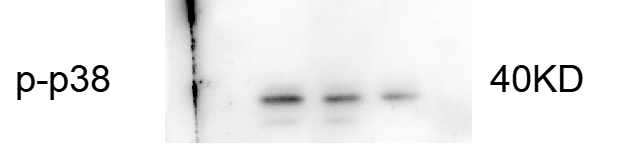

Supplement: Figure 4—figure supplement 1—source data 1. [file elife-107132-fig4-figsupp1-data1.zip › Figure 4-figure suplement 1 source data1-1/pp38.tif]

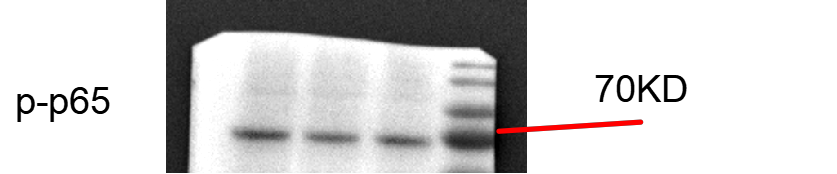

Supplement: Figure 4—figure supplement 1—source data 1. [file elife-107132-fig4-figsupp1-data1.zip › Figure 4-figure suplement 1 source data1-1/pp65.tif]

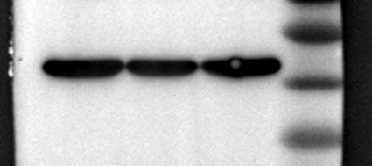

Supplement: Figure 4—figure supplement 1—source data 2. [file elife-107132-fig4-figsupp1-data2.zip › Figure 4-figure suplement 1 source data1/ba1.tif]

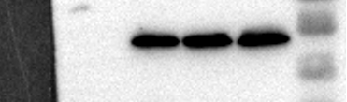

Supplement: Figure 4—figure supplement 1—source data 2. [file elife-107132-fig4-figsupp1-data2.zip › Figure 4-figure suplement 1 source data1/ba2.tif]

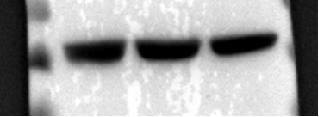

Supplement: Figure 4—figure supplement 1—source data 2. [file elife-107132-fig4-figsupp1-data2.zip › Figure 4-figure suplement 1 source data1/ba3.png]

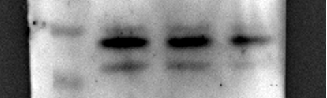

Supplement: Figure 4—figure supplement 1—source data 2. [file elife-107132-fig4-figsupp1-data2.zip › Figure 4-figure suplement 1 source data1/jnk.tif]

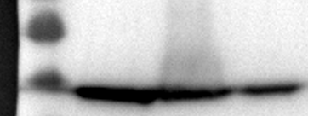

Supplement: Figure 4—figure supplement 1—source data 2. [file elife-107132-fig4-figsupp1-data2.zip › Figure 4-figure suplement 1 source data1/p38.tif]

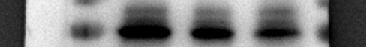

Supplement: Figure 4—figure supplement 1—source data 2. [file elife-107132-fig4-figsupp1-data2.zip › Figure 4-figure suplement 1 source data1/p65.tif]

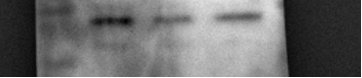

Supplement: Figure 4—figure supplement 1—source data 2. [file elife-107132-fig4-figsupp1-data2.zip › Figure 4-figure suplement 1 source data1/pjnk.tif]

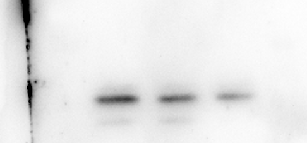

Supplement: Figure 4—figure supplement 1—source data 2. [file elife-107132-fig4-figsupp1-data2.zip › Figure 4-figure suplement 1 source data1/pp38.tif]

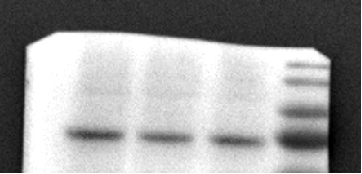

Supplement: Figure 4—figure supplement 1—source data 2. [file elife-107132-fig4-figsupp1-data2.zip › Figure 4-figure suplement 1 source data1/pp65.tif]

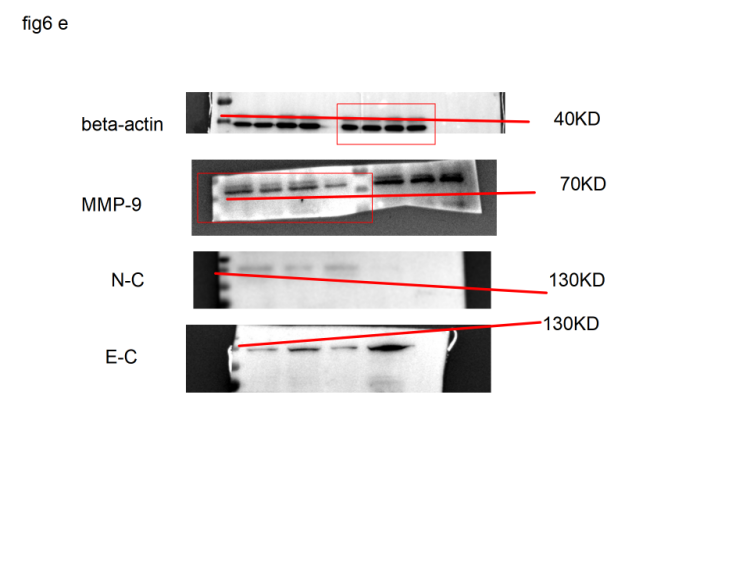

Supplement: Figure 6—source data 1. [file elife-107132-fig6-data1.zip › Figure 6 source data1-1/ALL.tif]

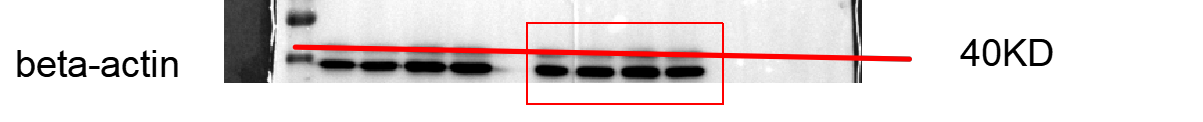

Supplement: Figure 6—source data 1. [file elife-107132-fig6-data1.zip › Figure 6 source data1-1/BA.tif]

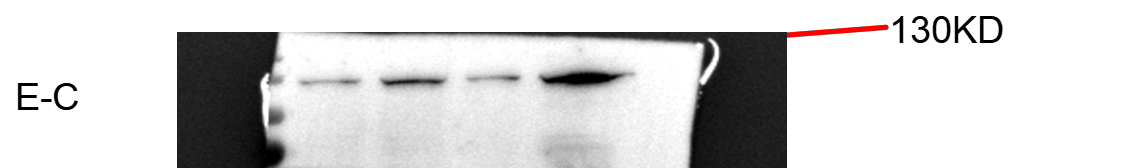

Supplement: Figure 6—source data 1. [file elife-107132-fig6-data1.zip › Figure 6 source data1-1/EC.tif]

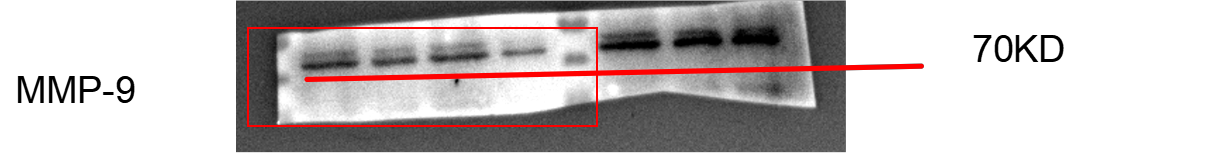

Supplement: Figure 6—source data 1. [file elife-107132-fig6-data1.zip › Figure 6 source data1-1/MMP9.tif]

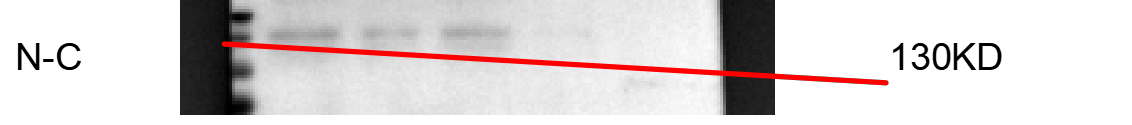

Supplement: Figure 6—source data 1. [file elife-107132-fig6-data1.zip › Figure 6 source data1-1/NC.tif]

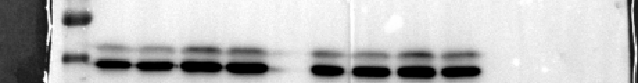

Supplement: Figure 6—source data 2. [file elife-107132-fig6-data2.zip › Figure 6 source data1/BA.tif]

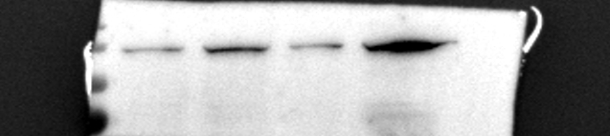

Supplement: Figure 6—source data 2. [file elife-107132-fig6-data2.zip › Figure 6 source data1/EC.tif]

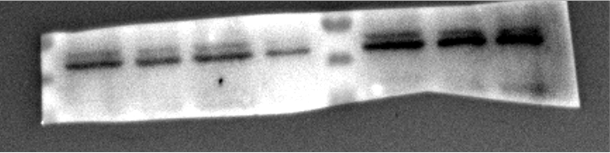

Supplement: Figure 6—source data 2. [file elife-107132-fig6-data2.zip › Figure 6 source data1/MMP9.tif]

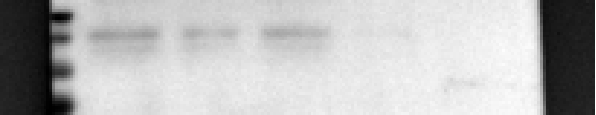

Supplement: Figure 6—source data 2. [file elife-107132-fig6-data2.zip › Figure 6 source data1/NC.tif]

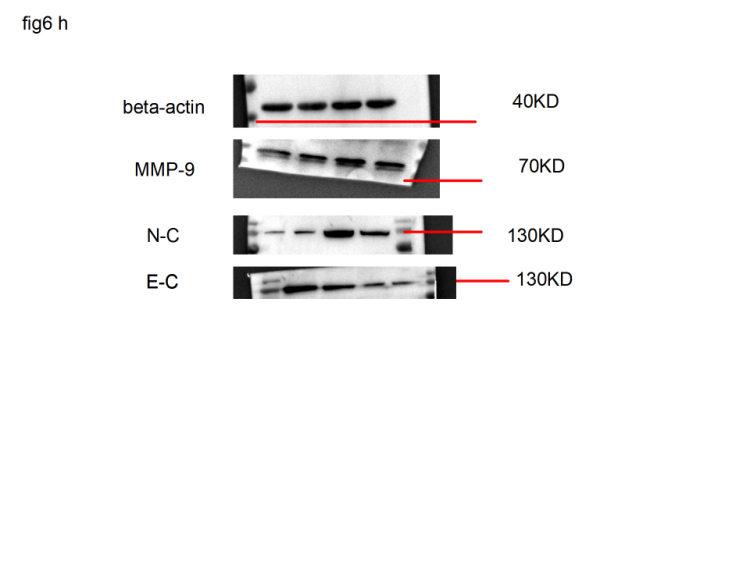

Supplement: Figure 6—source data 3. [file elife-107132-fig6-data3.zip › Figure 6 source data2-1/ALL.tif]

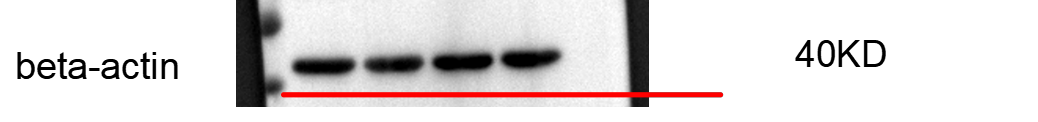

Supplement: Figure 6—source data 3. [file elife-107132-fig6-data3.zip › Figure 6 source data2-1/BA.tif]

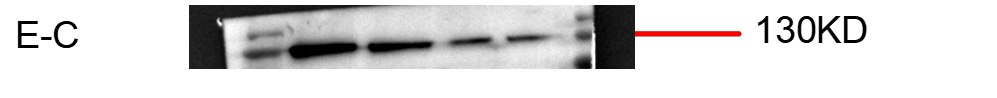

Supplement: Figure 6—source data 3. [file elife-107132-fig6-data3.zip › Figure 6 source data2-1/EC.tif]

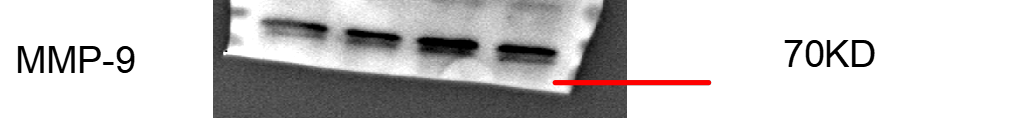

Supplement: Figure 6—source data 3. [file elife-107132-fig6-data3.zip › Figure 6 source data2-1/MMP9.tif]

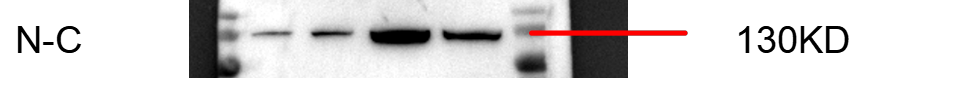

Supplement: Figure 6—source data 3. [file elife-107132-fig6-data3.zip › Figure 6 source data2-1/NC.tif]

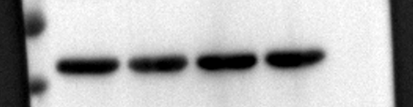

Supplement: Figure 6—source data 4. [file elife-107132-fig6-data4.zip › Figure 6 source data2/BA.tif]

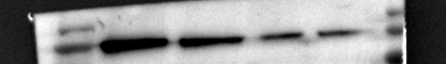

Supplement: Figure 6—source data 4. [file elife-107132-fig6-data4.zip › Figure 6 source data2/EC.tif]

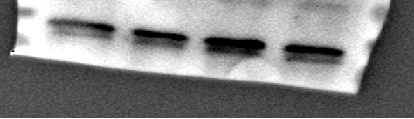

Supplement: Figure 6—source data 4. [file elife-107132-fig6-data4.zip › Figure 6 source data2/MMP9.tif]

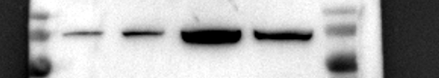

Supplement: Figure 6—source data 4. [file elife-107132-fig6-data4.zip › Figure 6 source data2/NC.tif]

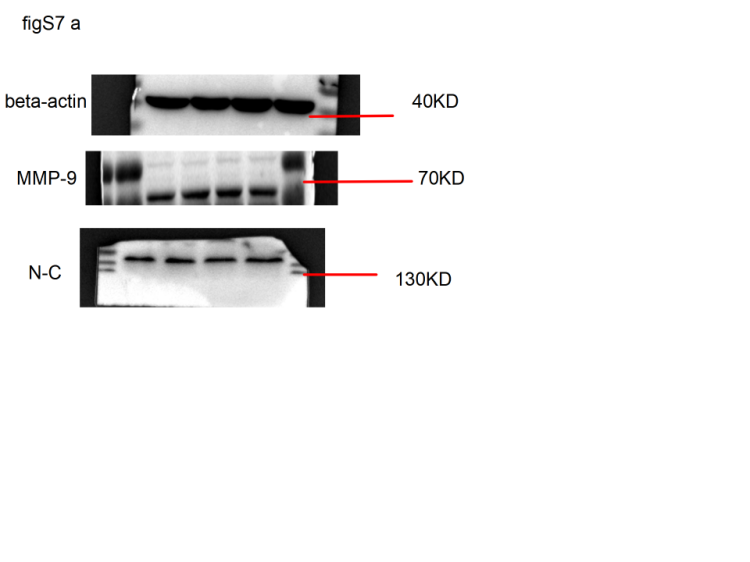

Supplement: Figure 6—figure supplement 1—source data 1. [file elife-107132-fig6-figsupp1-data1.zip › ALL.tif]
