# Supplementary figures and images for: RBMX2 links Mycobacterium bovis infection to epithelial–mesenchymal transition and lung cancer progression (part 2 of 2)
Source: eLife. 2025 Nov 24;14:RP107132. doi: 10.7554/eLife.107132 (PMC12643470; doi:10.7554/eLife.107132)

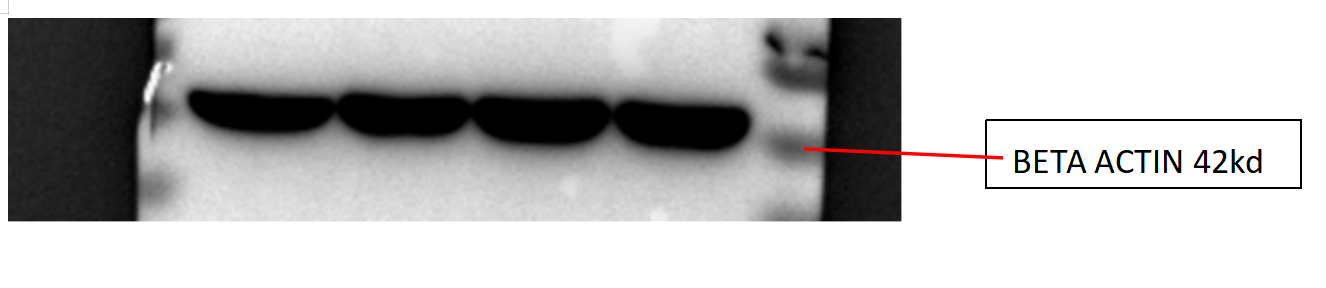

Supplement: Figure 6—figure supplement 1—source data 1. [file elife-107132-fig6-figsupp1-data1.zip › ba.png]

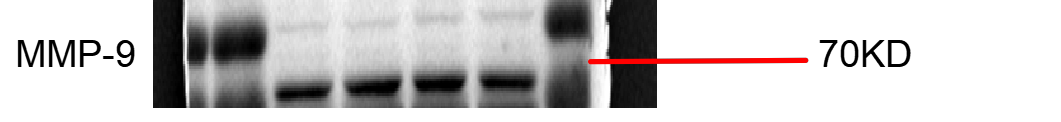

Supplement: Figure 6—figure supplement 1—source data 1. [file elife-107132-fig6-figsupp1-data1.zip › mmp9.tif]

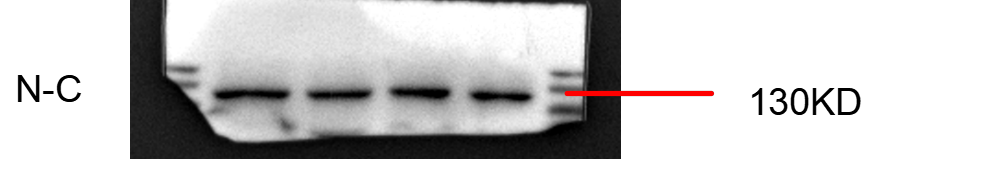

Supplement: Figure 6—figure supplement 1—source data 1. [file elife-107132-fig6-figsupp1-data1.zip › nc.tif]

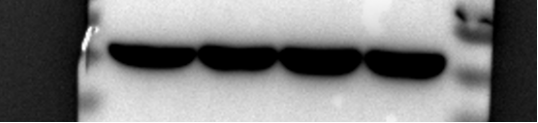

Supplement: Figure 6—figure supplement 1—source data 2. [file elife-107132-fig6-figsupp1-data2.zip › Figure 6-figure suplement 1 source data1/ba.tif]

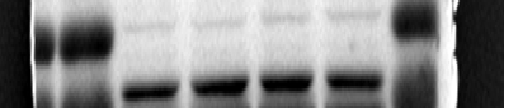

Supplement: Figure 6—figure supplement 1—source data 2. [file elife-107132-fig6-figsupp1-data2.zip › Figure 6-figure suplement 1 source data1/mmp9.tif]

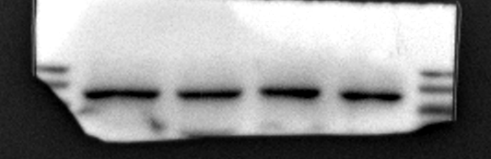

Supplement: Figure 6—figure supplement 1—source data 2. [file elife-107132-fig6-figsupp1-data2.zip › Figure 6-figure suplement 1 source data1/nc.tif]

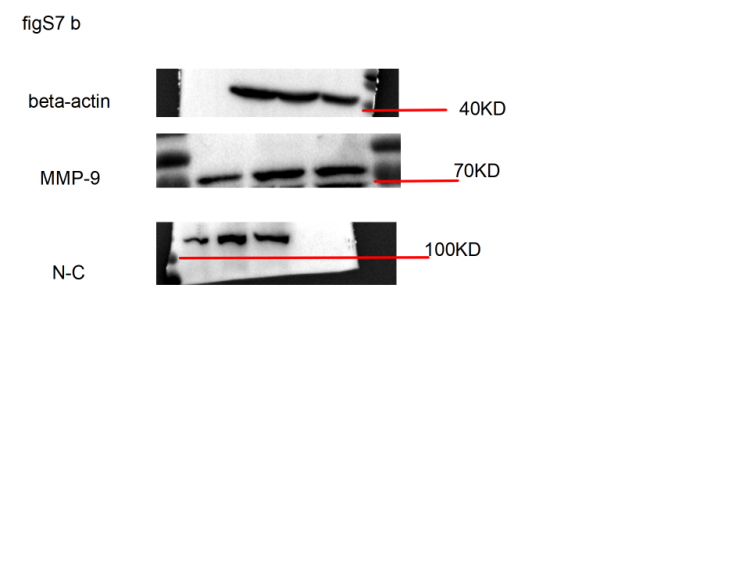

Supplement: Figure 6—figure supplement 1—source data 3. [file elife-107132-fig6-figsupp1-data3.zip › Figure 6-figure suplement 1 source data2-1/ALL.tif]

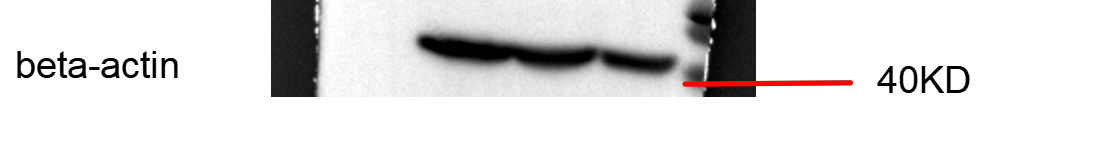

Supplement: Figure 6—figure supplement 1—source data 3. [file elife-107132-fig6-figsupp1-data3.zip › Figure 6-figure suplement 1 source data2-1/ba.tif]

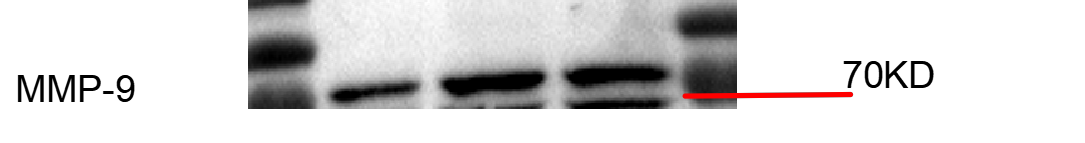

Supplement: Figure 6—figure supplement 1—source data 3. [file elife-107132-fig6-figsupp1-data3.zip › Figure 6-figure suplement 1 source data2-1/MMP9.tif]

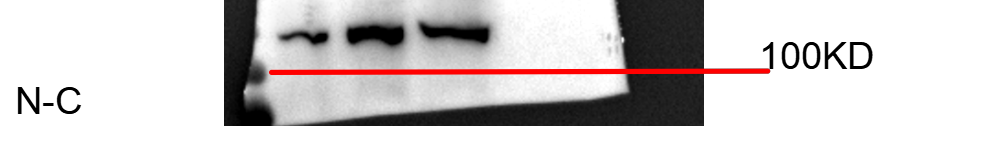

Supplement: Figure 6—figure supplement 1—source data 3. [file elife-107132-fig6-figsupp1-data3.zip › Figure 6-figure suplement 1 source data2-1/NC.tif]

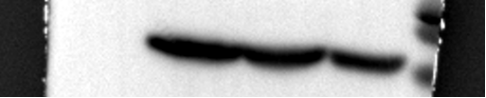

Supplement: Figure 6—figure supplement 1—source data 4. [file elife-107132-fig6-figsupp1-data4.zip › Figure 6-figure suplement 1 source data2/ba.tif]

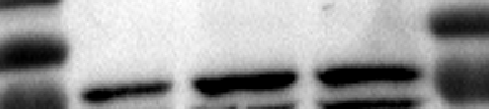

Supplement: Figure 6—figure supplement 1—source data 4. [file elife-107132-fig6-figsupp1-data4.zip › Figure 6-figure suplement 1 source data2/mmp9.tif]

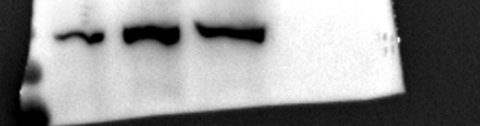

Supplement: Figure 6—figure supplement 1—source data 4. [file elife-107132-fig6-figsupp1-data4.zip › Figure 6-figure suplement 1 source data2/nc.tif]

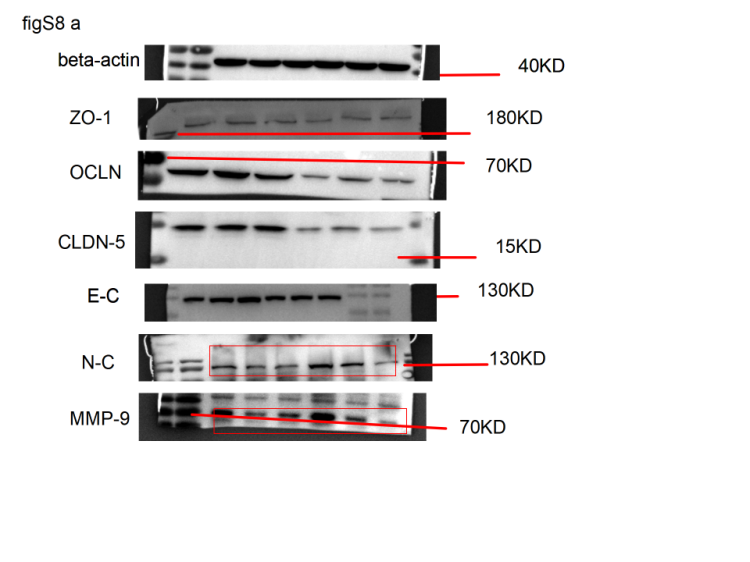

Supplement: Figure 6—figure supplement 2—source data 1. [file elife-107132-fig6-figsupp2-data1.zip › Figure 6-figure suplement 2 source data1-1/ALL.tif]

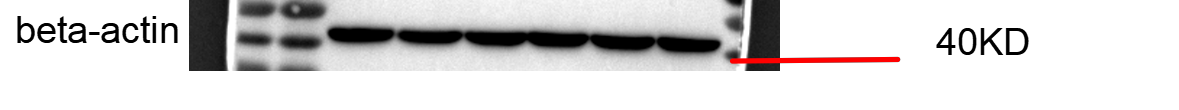

Supplement: Figure 6—figure supplement 2—source data 1. [file elife-107132-fig6-figsupp2-data1.zip › Figure 6-figure suplement 2 source data1-1/BA.tif]

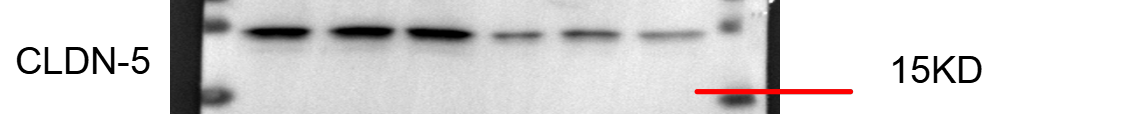

Supplement: Figure 6—figure supplement 2—source data 1. [file elife-107132-fig6-figsupp2-data1.zip › Figure 6-figure suplement 2 source data1-1/CLDN5.tif]

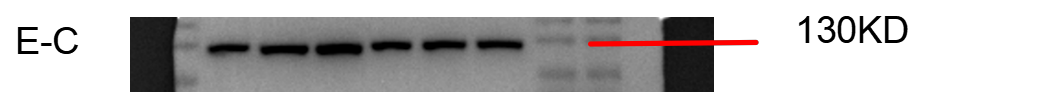

Supplement: Figure 6—figure supplement 2—source data 1. [file elife-107132-fig6-figsupp2-data1.zip › Figure 6-figure suplement 2 source data1-1/EC.tif]

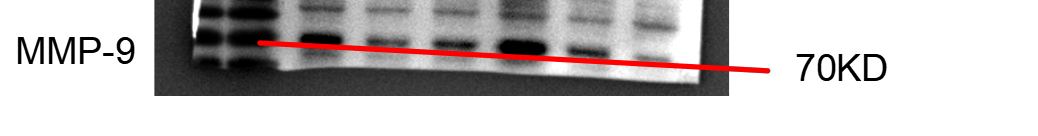

Supplement: Figure 6—figure supplement 2—source data 1. [file elife-107132-fig6-figsupp2-data1.zip › Figure 6-figure suplement 2 source data1-1/MMP9.tif]

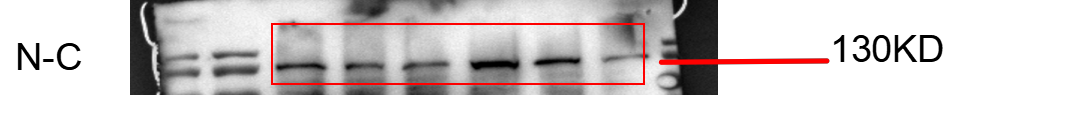

Supplement: Figure 6—figure supplement 2—source data 1. [file elife-107132-fig6-figsupp2-data1.zip › Figure 6-figure suplement 2 source data1-1/NC.tif]

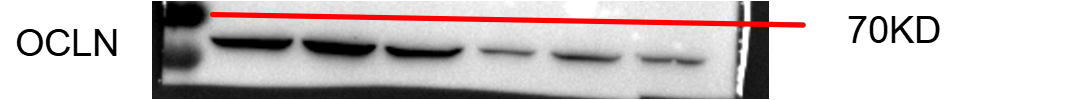

Supplement: Figure 6—figure supplement 2—source data 1. [file elife-107132-fig6-figsupp2-data1.zip › Figure 6-figure suplement 2 source data1-1/OCLN.tif]

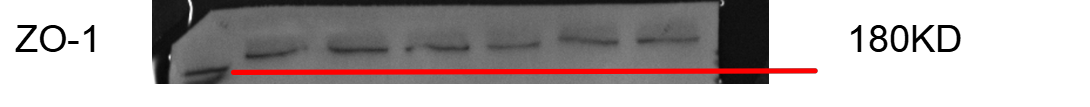

Supplement: Figure 6—figure supplement 2—source data 1. [file elife-107132-fig6-figsupp2-data1.zip › Figure 6-figure suplement 2 source data1-1/ZO1.tif]

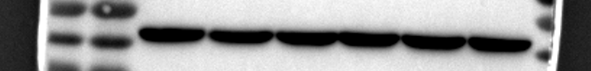

Supplement: Figure 6—figure supplement 2—source data 2. [file elife-107132-fig6-figsupp2-data2.zip › Figure 6-figure suplement 2 source data1/BA.tif]

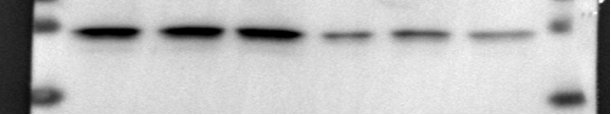

Supplement: Figure 6—figure supplement 2—source data 2. [file elife-107132-fig6-figsupp2-data2.zip › Figure 6-figure suplement 2 source data1/CLDN5.tif]

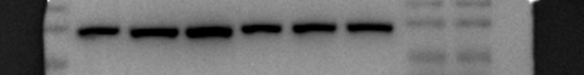

Supplement: Figure 6—figure supplement 2—source data 2. [file elife-107132-fig6-figsupp2-data2.zip › Figure 6-figure suplement 2 source data1/EC.tif]

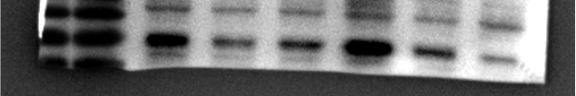

Supplement: Figure 6—figure supplement 2—source data 2. [file elife-107132-fig6-figsupp2-data2.zip › Figure 6-figure suplement 2 source data1/MMP9.tif]

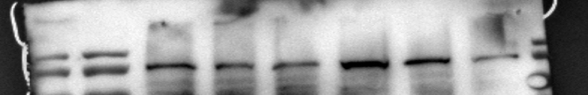

Supplement: Figure 6—figure supplement 2—source data 2. [file elife-107132-fig6-figsupp2-data2.zip › Figure 6-figure suplement 2 source data1/NC.png]

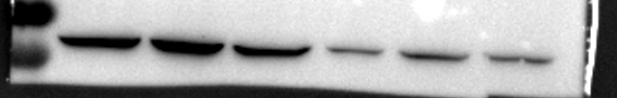

Supplement: Figure 6—figure supplement 2—source data 2. [file elife-107132-fig6-figsupp2-data2.zip › Figure 6-figure suplement 2 source data1/OCLN.tif]

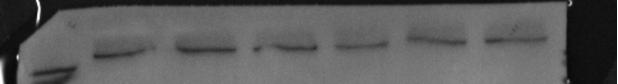

Supplement: Figure 6—figure supplement 2—source data 2. [file elife-107132-fig6-figsupp2-data2.zip › Figure 6-figure suplement 2 source data1/ZO1.tif]

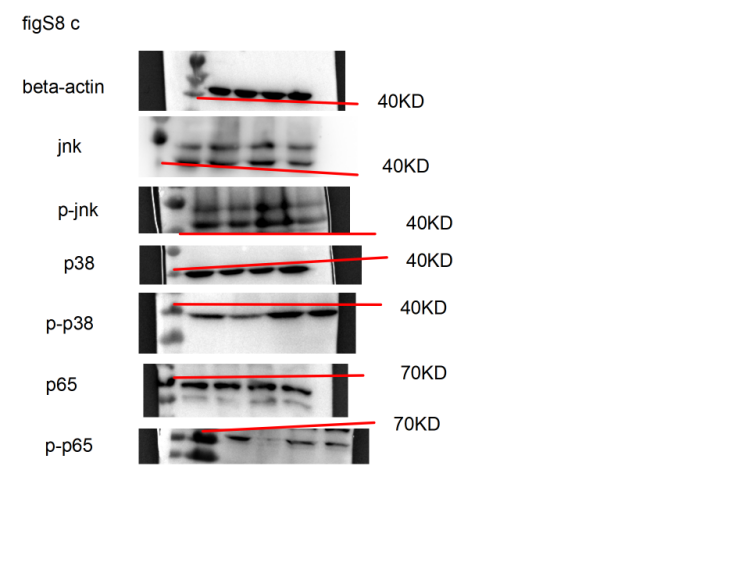

Supplement: Figure 6—figure supplement 2—source data 3. [file elife-107132-fig6-figsupp2-data3.zip › Figure 6-figure suplement 2 source data2-1/ALL.tif]

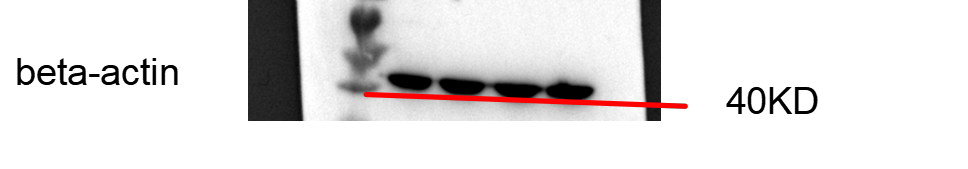

Supplement: Figure 6—figure supplement 2—source data 3. [file elife-107132-fig6-figsupp2-data3.zip › Figure 6-figure suplement 2 source data2-1/BA.tif]

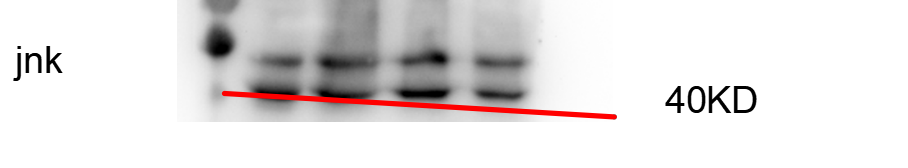

Supplement: Figure 6—figure supplement 2—source data 3. [file elife-107132-fig6-figsupp2-data3.zip › Figure 6-figure suplement 2 source data2-1/JNK.tif]

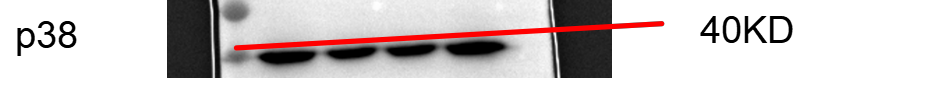

Supplement: Figure 6—figure supplement 2—source data 3. [file elife-107132-fig6-figsupp2-data3.zip › Figure 6-figure suplement 2 source data2-1/P38.tif]

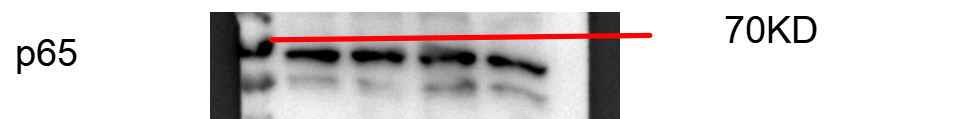

Supplement: Figure 6—figure supplement 2—source data 3. [file elife-107132-fig6-figsupp2-data3.zip › Figure 6-figure suplement 2 source data2-1/P65.tif]

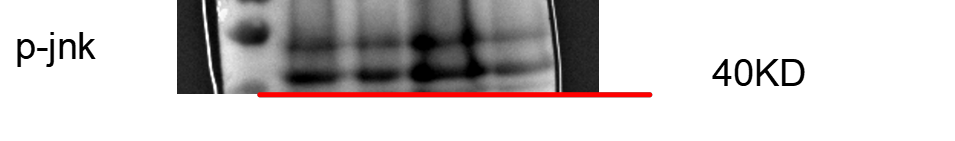

Supplement: Figure 6—figure supplement 2—source data 3. [file elife-107132-fig6-figsupp2-data3.zip › Figure 6-figure suplement 2 source data2-1/PJNK.tif]

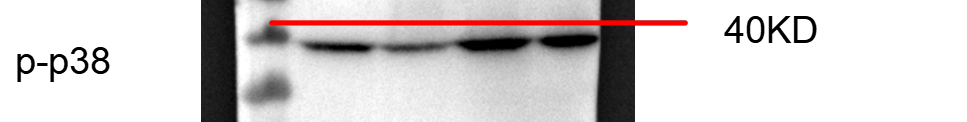

Supplement: Figure 6—figure supplement 2—source data 3. [file elife-107132-fig6-figsupp2-data3.zip › Figure 6-figure suplement 2 source data2-1/PP38.tif]

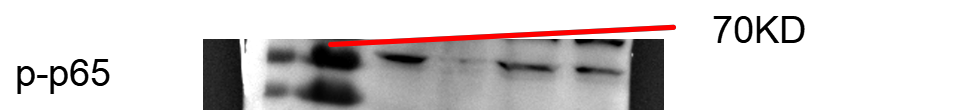

Supplement: Figure 6—figure supplement 2—source data 3. [file elife-107132-fig6-figsupp2-data3.zip › Figure 6-figure suplement 2 source data2-1/PP65.tif]

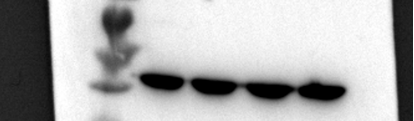

Supplement: Figure 6—figure supplement 2—source data 4. [file elife-107132-fig6-figsupp2-data4.zip › Figure 6-figure suplement 2 source data2/BA.tif]

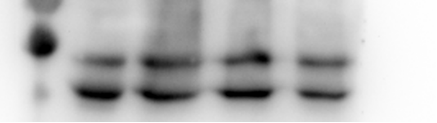

Supplement: Figure 6—figure supplement 2—source data 4. [file elife-107132-fig6-figsupp2-data4.zip › Figure 6-figure suplement 2 source data2/JNK.tif]

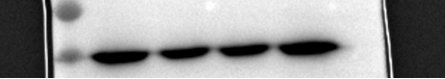

Supplement: Figure 6—figure supplement 2—source data 4. [file elife-107132-fig6-figsupp2-data4.zip › Figure 6-figure suplement 2 source data2/P38.png]

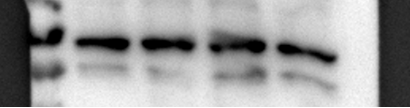

Supplement: Figure 6—figure supplement 2—source data 4. [file elife-107132-fig6-figsupp2-data4.zip › Figure 6-figure suplement 2 source data2/P65.tif]

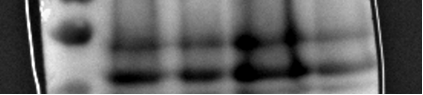

Supplement: Figure 6—figure supplement 2—source data 4. [file elife-107132-fig6-figsupp2-data4.zip › Figure 6-figure suplement 2 source data2/PJNK.tif]

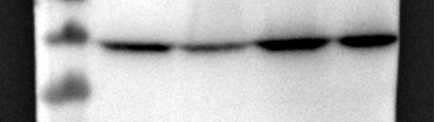

Supplement: Figure 6—figure supplement 2—source data 4. [file elife-107132-fig6-figsupp2-data4.zip › Figure 6-figure suplement 2 source data2/PP38.png]

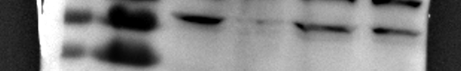

Supplement: Figure 6—figure supplement 2—source data 4. [file elife-107132-fig6-figsupp2-data4.zip › Figure 6-figure suplement 2 source data2/PP65.tif]

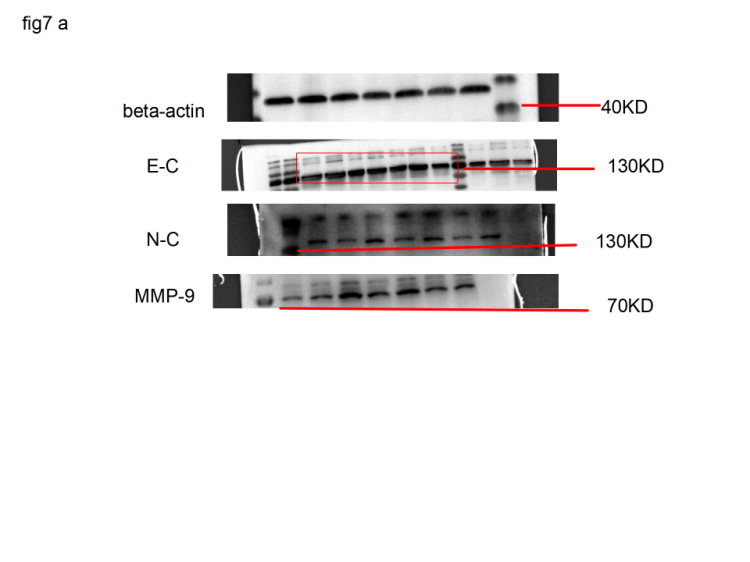

Supplement: Figure 7—source data 1. [file elife-107132-fig7-data1.zip › Figure 7 source data1-1/ALL.tif]

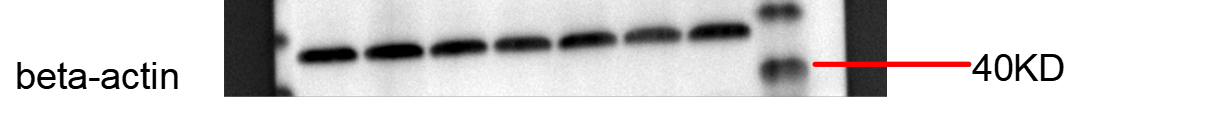

Supplement: Figure 7—source data 1. [file elife-107132-fig7-data1.zip › Figure 7 source data1-1/BA.tif]

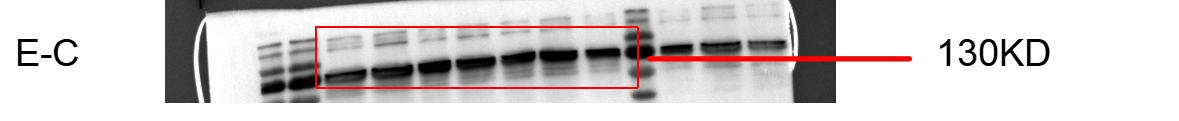

Supplement: Figure 7—source data 1. [file elife-107132-fig7-data1.zip › Figure 7 source data1-1/EC.tif]

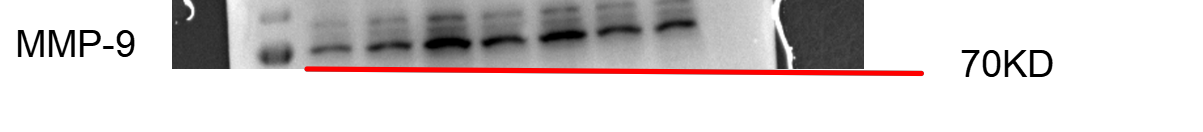

Supplement: Figure 7—source data 1. [file elife-107132-fig7-data1.zip › Figure 7 source data1-1/MMP9.tif]

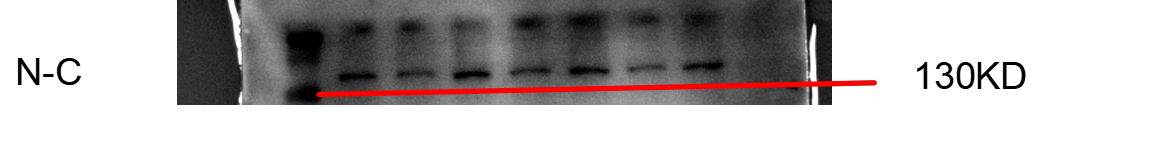

Supplement: Figure 7—source data 1. [file elife-107132-fig7-data1.zip › Figure 7 source data1-1/NC.tif]

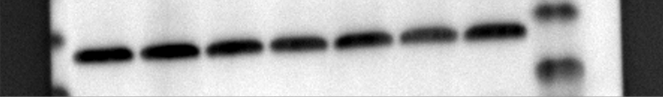

Supplement: Figure 7—source data 2. [file elife-107132-fig7-data2.zip › Figure 7 source data1/BA.tif]

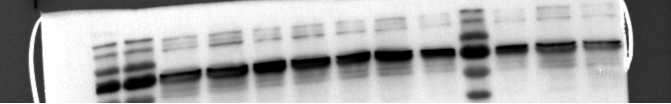

Supplement: Figure 7—source data 2. [file elife-107132-fig7-data2.zip › Figure 7 source data1/EC.tif]

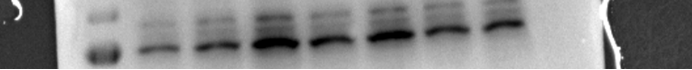

Supplement: Figure 7—source data 2. [file elife-107132-fig7-data2.zip › Figure 7 source data1/MMP9.tif]

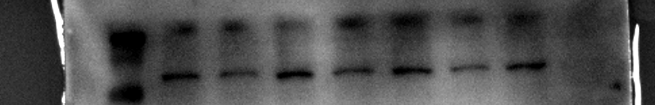

Supplement: Figure 7—source data 2. [file elife-107132-fig7-data2.zip › Figure 7 source data1/NC.tif]

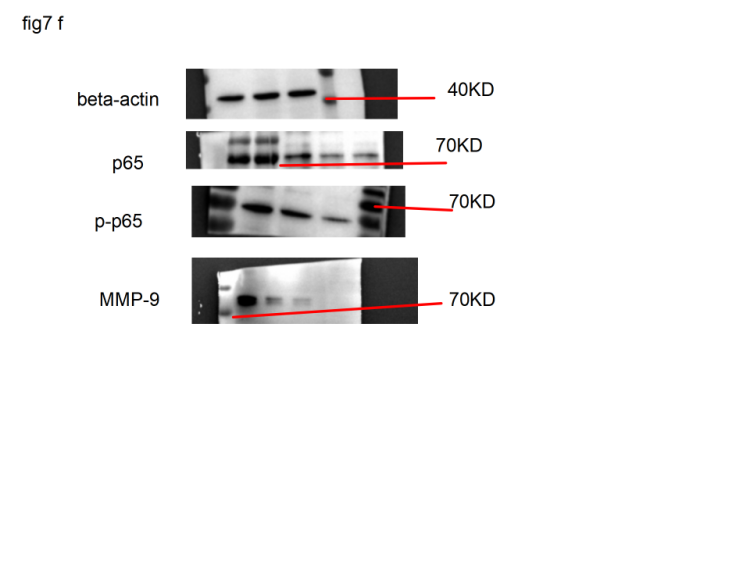

Supplement: Figure 7—source data 3. [file elife-107132-fig7-data3.zip › Figure 7 source data2-1/ALL.tif]

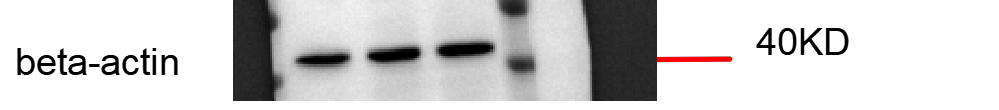

Supplement: Figure 7—source data 3. [file elife-107132-fig7-data3.zip › Figure 7 source data2-1/BA.tif]

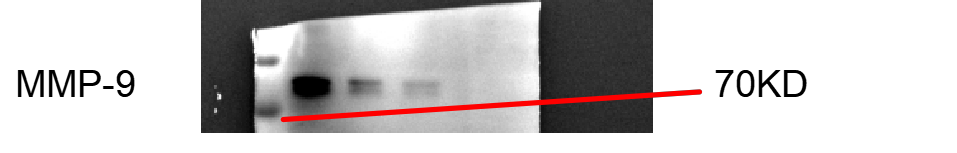

Supplement: Figure 7—source data 3. [file elife-107132-fig7-data3.zip › Figure 7 source data2-1/MMP9.tif]

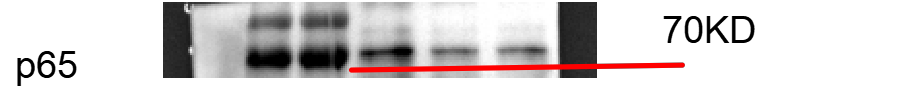

Supplement: Figure 7—source data 3. [file elife-107132-fig7-data3.zip › Figure 7 source data2-1/P65.tif]

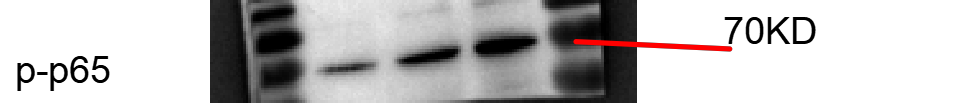

Supplement: Figure 7—source data 3. [file elife-107132-fig7-data3.zip › Figure 7 source data2-1/PP65.tif]

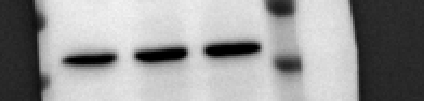

Supplement: Figure 7—source data 4. [file elife-107132-fig7-data4.zip › Figure 7 source data2/BA.tif]

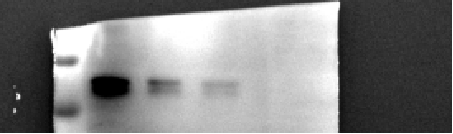

Supplement: Figure 7—source data 4. [file elife-107132-fig7-data4.zip › Figure 7 source data2/MMP9.tif]

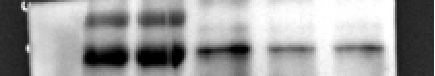

Supplement: Figure 7—source data 4. [file elife-107132-fig7-data4.zip › Figure 7 source data2/P65.tif]

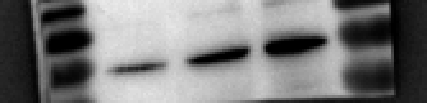

Supplement: Figure 7—source data 4. [file elife-107132-fig7-data4.zip › Figure 7 source data2/PP65.tif]

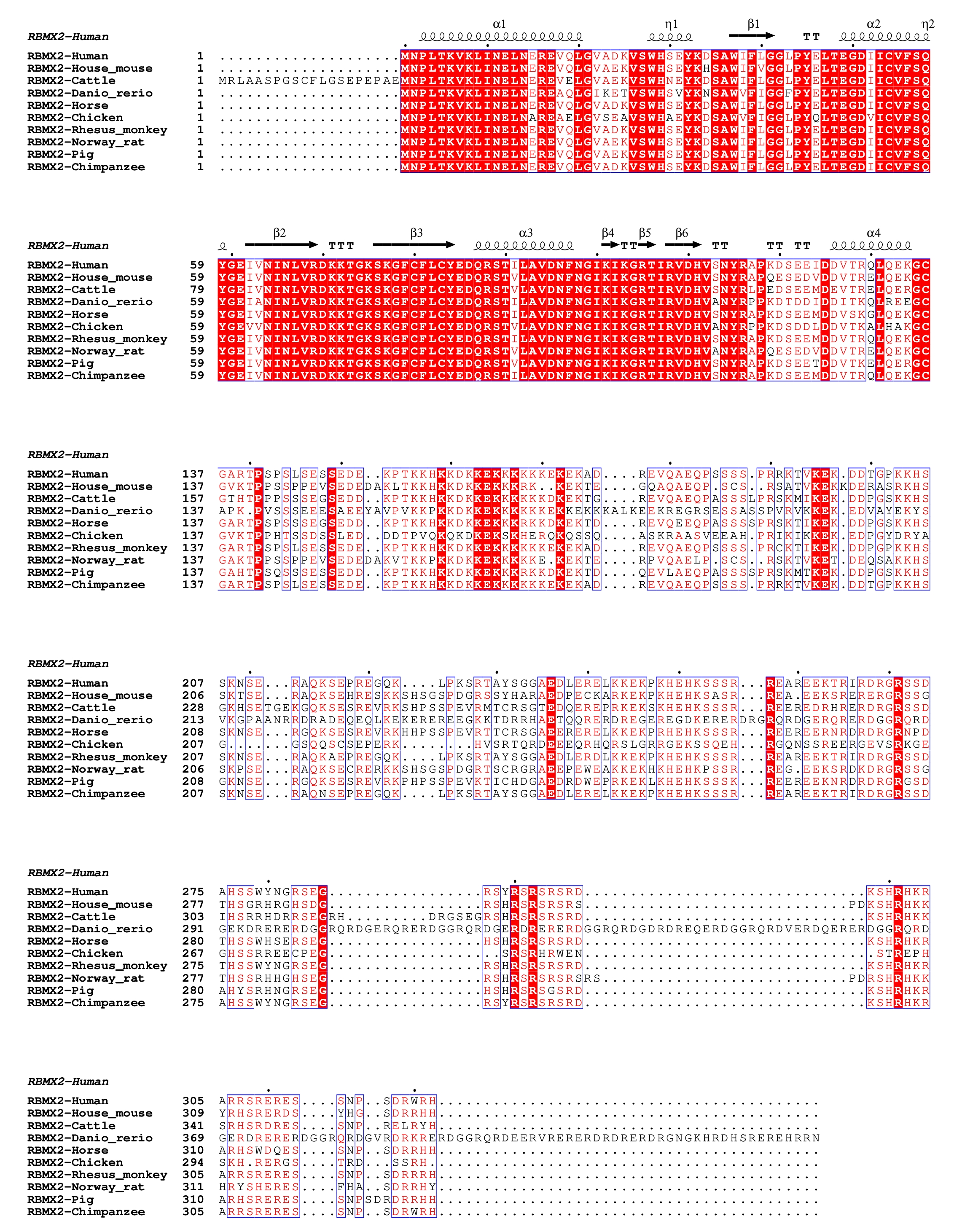

Supplement: Supplementary file 1. [file elife-107132-supp1.zip › Supplementary Table1/Supplementary Table1.tif]
